# Impact on polytrauma patient prehospital care during the first wave of the COVID-19 pandemic: a cross-sectional study

**DOI:** 10.1007/s00068-021-01748-3

**Published:** 2021-07-30

**Authors:** Silvia Solà-Muñoz, Oriol Yuguero, Youcef Azeli, Guillermo Roig, José Antonio Prieto-Arruñada, Jaume Español, Jorge Morales-Álvarez, Manuel Muñoz, Juan José Verge, Xavier Jiménez-Fàbrega

**Affiliations:** 1 Sistema d’Emergències Mèdiques de Catalunya, Barcelona, Spain; 2 Red de Investigación Emergencias Prehospitalarias (RINVEMER), Madrid, Spain; 3 Transversal Research Group on Emergencies. IRBLLEIDA, AVda. Rovira Roure 80, 25198 Lleida, Spain; 4 Faculty of Medicine, University of Lleida, Lleida, Spain; 5 Institut d’Investigació Sanitari Pere i Virgili (IISPV), Tarragona, Spain; 6 Hospital Universitari Sant Joan de Reus, Tarragona, Spain

**Keywords:** Polytrauma, SARS-CoV-2, Prehospital, Injury, Timely life-saving interventions, Code

## Abstract

**Background:**

The extraordinary situation caused by the onset of COVID-19 has meant that at prehospital level, the number of treatments, profile and time taken to respond for treating time-dependent pathologies has been greatly affected. However, it is not known whether the prehospital profile of polytrauma patients (PTP) has been affected.

**Objective:**

To determine differences in the epidemiological characteristics and the clinical variables of prehospital polytrauma patients during the first wave of the COVID-19 pandemic in Catalonia.

**Methodology:**

Analytical cross-sectional study. The number of prehospital activations and the clinical and epidemiological characteristics of polytrauma patients attended by the Emergency Medical System (EMS) of Catalonia, were compared for the period between 15 February and 15 May 2020 and the same period in the previous year. Priorities 0 and 1 are assigned to the most severely injured patients. An analysis was conducted using logistic regression and nonparametric tests.

**Results:**

3023 patients were included. During the 2019 study period, 2045 (67.6%) patients were treated; however, during the pandemic period, 978 (32.4%) patients were treated, representing a 52% decrease (*p* = 0.002). The percentage of patients presenting priority 1 was higher during the pandemic period [240 (11.7%) vs 146 (14.9%), *p* = 0.032]. The percentage of priority 0 and 1 patients attended by a basic life support unit increased [201 (9.8%) vs 133 (13.6%), *p* = 0.006]. The number of traffic accidents decreased from 1211 (59.2%) to 522 (53.4%) and pedestrian-vehicle collisions fell from 249 (12.2%) to 92 (9.4%). Regarding weapon-related injuries and burns, there was an increase in the number of cases [43 (2.1%) vs 41 (4.2%), and 15 (0.7%) vs 22 (2.2%), *p* = 0.002 and *p* < 0.001, respectively]. Hospital mortality remained unchanged (3.9%).

**Conclusions:**

During the first wave of the pandemic, the number of polytrauma patients decreased and there was a change in the profile of severity and type of accident.

## Background

In December 2019, an infectious outbreak of unknown aetiology was detected in Wuhan, Hubei Province, China. It has been identified as the agent that caused the outbreak of a novel coronavirus, SARS-CoV-2 [1]. On 11 March 2020, the World Health Organization (WHO) declared the outbreak of coronavirus SARS-CoV-2 an international public health emergency affecting more than 4.5 million people, having caused more than 14,510 deaths [2]. By early July 2020, Spain had recorded more than 249,000 cases and 28,000 deaths [3].

The extraordinary health situation caused by the pandemic has changed many routine healthcare criteria, protocols and strategies. The gap between the demand for and the availability of resources, the need for healthcare teams to self-protect and the logistical difficulties resulting from the saturation of the emergency care system have had a major impact on many healthcare processes in emergency departments [4]. The intensity of the outbreak in certain regions and overwhelmed emergency services and critical care units may affect healthcare provided to other pathologies. The system has been under such strain that it has been difficult to offer a similar response to the period prior to the pandemic, even for pathologies with well-established circuits such as the treatment of time-dependent pathologies like acute myocardial infarction [5] or stroke [6].

Difficulties at coordination centres, call overload, patients’ fear of contagion, and the essential use of personal protective equipment, have come to alter some emergency medical system (EMS) procedures [7–9] including time-dependent pathology circuits: activation codes [10].

Since 2011, in Catalonia attention to severe trauma patients has been regulated by a specific procedure involving prehospital care and emergency departments of hospitals in Catalonia [11, 12]. The process involves a high degree of coordination and communication between the healthcare team, the health coordination centre and the referral hospital. Having identified the severity of the patient, he or she is prioritized according to specific treatment needs and is transferred to the optimum centre to provide a definitive response.

The impact of the pandemic on healthcare for time-dependent pathologies and on the chain of healthcare given to the polytrauma patient is not well established. However, new studies have emerged in recent weeks [13]. Given the declared state of emergency and restricted mobility, it was considered timely, as an objective of the study, to analyse the differences in the epidemiological and clinical profiles of prehospital polytrauma patients between the months of February and May 2020, compared to the same period for the previous year.

## Methods

### Study design and setting

A cross-sectional, comparative, analytical study was conducted using the EMS polytrauma data base. Patient inclusion spanned two periods: (1) pre-COVID-19: from 15 February to 15 May 2019, and (2) COVID-19 period: from 15 February to 15 May 2020. The study was conducted throughout the territory of Catalonia, which has a population of 7,727,029 inhabitants. Catalonia has a surface area of 32,000 km^2^ and an average density of 239 inhabitants/km^2^. The most populated areas are the urban areas corresponding to the region’s four provincial capitals, which account for 35% of the total population. Medical emergencies occurring outside the hospital setting are attended to only by the Sistema d’Emergències Mèdiques de Catalunya, the EMS of the public health system providing coverage throughout 100% of the territory. The study has been approved by the Ethics Committee of Institut d'Investigació Sanitària Pere Virgili (Ref: 148/2020).

Trauma patients are classified according to different levels of priority for attention. Priority 0 is assigned to critical patients who are physiologically compromised (difficulty breathing, low blood pressure, alternating levels of consciousness) and require transferring to centres equipped to receive severely injured patients. Priority 1 is based on anatomical criteria (penetrating wounds to the head, neck, thorax or abdomen, open skull fracture, flail chest, fracture of two or more long bones or pelvis, proximal amputation at the ankle/wrist, suspected medullary lesion, catastrophic limb and severe burns). Priority 2 is established based on the high-energy injury mechanism (high speed, cabin deformity, a death at the scene, a fall from a great height), and priority 3 is triggered according to the patient’s medical records (administered anticoagulants, known renal failure, pregnancy).

### Inclusion and exclusion criteria

All polytrauma patients attended primarily by the EMS were included consecutively. Patients transferred from one hospital to another and those for whom the code was activated from hospital level were excluded.

### Data collection

The doctor or nurse in charge of the advanced life support unit or the health care technician of the basic life support unit who attended to the patient, collected the data prospectively in a computer application designed for this purpose which forms part of the digitized clinical report. Data on healthcare pathway times and referral hospital were automatically collected by the coordination centre.

Data collected included the number of patients treated, epidemiological variables (gender, age, municipality where the service was provided, type of medical care unit set in motion) and variables related to healthcare pathway times: (a) medical coordination centre management times, including alert, allocation and activation of resources, and (b) patient treatment times, from mobilizing the resource to patient transfer to hospital. Finally, the characteristics for the management of polytrauma care were collected: type of accident, part of the body affected, haemodynamic status (by measuring blood pressure), breathing (assessing the need for advanced airway management), neurological status (recorded using the Glasgow Coma Scale), referral hospital, prehospital diagnosis, prehospital treatment and priority. Data for primary treatment and hospital mortality were also collected.

### Statistical analysis

Calculation of sample size to detect a 10% decrease in the number of services, considering a power of 85% and an alpha risk of 5%, was of 913 patients per group. In the descriptive analysis, the qualitative variables are described as absolute frequency and percentages with their 95% confidence interval (CI95%) and quantitative variables as mean, standard deviation, median and interquartile range. The Shapiro–Wilk test was performed to check the normality of the quantitative variables. Logistic regression was used for the comparative study of the qualitative variables, and the Student’s *t* test and the nonparametric Mann–Whitney test were used to analyse the quantitative variables. All statistical analyses were performed using the SPSS statistical package (version 24.0, SPSS Inc., Chicago, Illinois), and a p value of *p* < 0.05 was considered statistically significant.

## Results

**Fig. 1:**
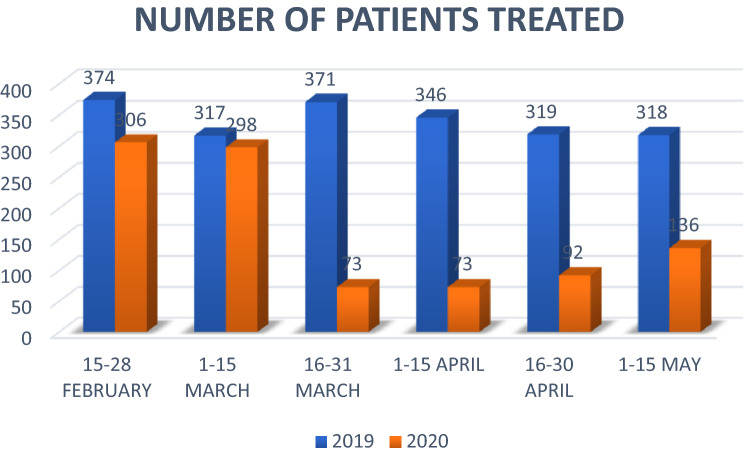
Prehospital activations of the PTP Code (polytrauma patient code)

The study included 3023 polytrauma patients treated by the EMS in Catalonia. During the 2019 study period, 2045 (67.6%) patients were treated; however, during the pandemic period, 978 (32.4%) patients were treated, representing a 52% decrease (*p* = 0.002). Figure 1 shows a reduction of patients treated per fortnight that is most pronounced from 15 March 2020 onwards, coinciding with the start of lockdown on 14 March.

Regarding the epidemiological characteristics of the sample, 71.5% were male and the most common age group was 31–50 years (37.7%) with a mean age of 40.3 (SD 18.9 years), as set out in Table 1. Of the entire sample, 8.1% were under 18 years of age. There were no changes in 2020 in terms of mean age or gender. The number of cases was proportional to the regional population, i.e., 60% originated in the Barcelona health region, 10.4% in Girona, 9% in Tarragona, and 4.5% in Lleida.

**Table 1:** Epidemiological characteristics of the sample

	Global (*n* = 3023; 100%)		Year 2019 (*n* = 2045; 67.6%)		Year 2020 (*n* = 978; 32.4%)		OR (CI 95%)	p*
	*N*	%	*N*	%	*N*	%		
Male gender	2161	71.50%	1452	71.00%	709	72.50%	1.02(0.91–0.15)	0.729
Age								
< 18 years	245	8.10%	168	8.20%	77	7.90%	0.90 (0.69–1.19)	0.479
18–30 years	682	22.60%	466	22.80%	216	22.10%	0.97 (0.81–1.16)	0.731
31–50 years	1139	37.70%	751	36.70%	388	39.70%	1.08 (0.93–1.24)	0.294
> 50 years	957	31.70%	660	32.30%	297	30.40%	0.94 (0.80–1.10)	0.446
Health región								
Barcelona city	1817	60.10%	1226	60.00%	591	60.40%	1.01 (0.89–1.14)	0.9
Girona	319	10.60%	221	10.70%	100	10.20%	0.95 (0.74–1.21)	0.662
Central Catalonia	290	9.50%	186	9.10%	103	10.50%	1.16 (0.90–1.49)	0.255
Camp de Tarragona	272	9.00%	193	9.40%	79	8.10%	0.86 (0.65–1.12)	0.263
Lleida	135	4.50%	98	4.80%	37	3.80%	0.79 (0.54–1.16)	0.229
Terres de l'Ebre	118	3.90%	72	3.50%	45	4.60%	1.31 (0.89–1.91)	0.168
Alt Pirineu and Aran	72	2.40%	49	2.40%	23	2.40%	0.98 (0.50–1.62)	0.942
Resource type assigned								
Air ALS	83	2.50%	53	2.60%	30	3.10%	1.18 (0.75–1.86)	0.467
Ground ALS	995	33.00%	703	34.40%	292	29.90%	0.81 (0.69–0.96)	0.014*
BLS	1945	64.30%	1289	63.00%	656	67.70%	1.23 (1.05–1.45)	0.011*

*OR* odds ratio, *air ALS* air advanced life support, *ground ALS* ground advanced life support, *BLS* basic life support; p*: *p* < 0.05

Bold means statistical significance (p<0,05)

Road traffic accidents decreased abruptly at the start of lockdown (14 March 2020), falling by 22.7% (61.8% before and 39.1% after lockdown; *p* < 0,001). However, unlike road traffic accidents, vehicle–pedestrian collisions decreased throughout the pandemic period (*p* = 0.043). An increase in accidents caused by weapons (*p* = 0.031) and burns (*p* = 0.007) was also noted, especially in the home in 2020 (24.3% in 2019 vs 46.1% in 2020; *p* = 0.016). Finally, a rise in traumatic falls was noted exclusively after the onset of lockdown.

Table 2 shows the types of accident in the total sample and the different periods of the study. The number of traffic accidents decreased from 1211 (59.2%) to 522 (53.4%), and pedestrian-vehicle collisions fell from 249 (12.2%) to 92 (9.4%). Regarding injuries caused by weapons and burns, there was an increase in the percentage of cases [43 (2.1%) vs 41 (4.2%) and 15 (0.7% vs 22 (2.2%), *p* = 0.002 and *p* < 0.001, respectively].

**Table 2:** Characteristics of cases of PTP code analysed

	Global (*n* = 3023; 100%)		Year 2019 (*n* = 2045; 67.6%)		Year 2020 (*n* = 978; 32.4%)		OR (CI 95%)	p*
	*N*	%	*N*	%	*N*	%		
PTP code priority								
0	223	7.4%	148	7.2%	75	7.7%	1.06 (0.79–1.41)	0.694
1	386	12.7%	240	11.7%	146	14.9%	1.27 (1.02–1.58)	0.032*
2	2300	76.1%	1575	77.0%	725	74.1%	0.96 (0.86–1.08)	0.520
3	114	3.8%	82	4.0%	32	3.3%	0.82 (0.54–1.24)	0.338
PTP priority code according to responsible resource								
BLS P0 and P1	334	11.0%	195	9.5%	139	14.2%	1.49 (1.18–1.88)	< 0.001*
ALS P0 and P1	275	9.1%	193	9.4%	82	8.4%	0.89 (0.68–1.15)	0.389
BLS P2 and P3	1611	53.3%	1084	53.0%	527	53.9%	1.02 (0.89–1.16)	0.803
ALS P2 and P3	803	26.6%	573	28.1%	230	23.5%	0.84 (0.71–0.99)	0.044*
Type of accident								
Road traffic accident	1735	57.4%	1211	59.2%	522	53.4%	0.90 (0.79–1.02)	0.111
Vehicle–pedestrian collision	341	11.3%	249	12.2%	92	9.4%	0.77 (0.60–0.99)	0.043*
Fall	703	23.3%	454	22.2%	249	25.5%	1.15 (0.97–1.36)	0.119
Cold weapon/firearm	82	2.7%	43	2.1%	41	4.2%	1.99 (1.29–3.08)	0.002*
Other aggression	25	0.8%	14	0.7%	11	1.1%	1.64 (0.74–3.63)	0.220
Burns	37	1.2%	15	0.7%	22	2.2%	3.07 (1.58–5.94)	< 0.001*
Others	100	3.3%	59	2.9%	41	4.2%	1.45 (0.97–2.18)	0.071
Part of body affected								
Without injuries	100	3.3%	69	3.4%	31	3.2%	0.94 (0.61–1.43)	0.760
Head	1136	37.6%	761	37.2%	375	38.3%	1.03 (0.90–1.18)	0.708
Face	478	15.8%	331	16.2%	147	15.0%	0.92 (0.76–1.13)	0.449
Neck	458	15.2%	316	15.5%	142	14.5%	0.94 (0.76–1.15)	0.527
Thorax	681	22.5%	476	23.3%	205	21.0%	0.90 (0.75–1.07)	0.218
Abdomen	262	8.7%	188	9.2%	74	7.6%	0.89 (0.74–1.06)	0.215
Pelvis	266	8.8%	166	8.1%	100	10.2%	1.25 (0.97–1.62)	0.081
Spine	521	17.2%	338	16.5%	183	18.7%	1.13 (0.93–1.36)	0.211
Limbs	1606	53.1%	1077	52.7%	529	54.1%	1.02 (0.91–1.15)	0.707
External injuries	130	4.3%	87	4.3%	43	4.4%	1.03 (0.71–1.49)	0.874
Respiratory condition								
Invasive airway management	179	5.9%	117	5.7%	62	6.3%	1.11 (0.81–1.52)	0.526
Difficulty breathing	100	3.3%	70	3.4%	30	3.1%	0.90 (0.58–1.38)	0.621
Normal	2744	90.8%	1858	90.9%	886	90.6%	0.99 (0.89–1.11)	0.959
Haemodynamic status								
No pulse or SBP < 50 mmHg	186	6.1%	125	6.1%	61	6.2%	1.02 (0.74–1.40)	0.900
SBP 50–90 mmHg	157	5.2%	106	5.2%	51	5.2%	1.00 (0.71–1.42)	0.973
SBP > 90 mmHg	2680	88.7%	1814	88.7%	866	88.5%	0.99 (0.89–1.12)	0.975
Neurological status								
GCS 15	2464	81.5%	1673	81.8%	791	80.9%	0.99 (0.88–1.11)	0.844
GCS 9–14	395	13.1%	264	12.9%	131	13.4%	1.04 (0.83–1.30)	0.746
GCS ≤ 8	164	5.4%	108	5.3%	56	5.7%	1.08 (0.77–1.51)	0.633

*OR* odds ratio, *BLS* basic life support, *ALS* advanced life support, *P0* priority 0, *P1* priority 1, *P2* priority 2, *P3* priority 3, *SBP* systolic blood pressure, *mmHg* millimetres of mercury, *GCS* Glasgow Coma Scale. p*:* p* < 0.05

Bold means statistical significance (p<0,05)

### Trauma management

Regarding the resource performing the initial intervention, there was a significant decrease in the number of services provided by ground advanced life support (ALS) units [703 (34.4%) vs 292 (29.9%), *p* = 0.014]; conversely, the activities of basic life support (BLS) units increased by 4.7% (*p* = 0.011) (Table 1). BLS units dealt with more severe patients, with a significant increase in the percentage of services classified as priority 0 and 1 during the pandemic period [195 (9.5%) vs 139 (14.2%), *p* < 0.001] as set out in Table 2. However, ALS units decreased their activity with priority 2 and 3 patients by 4.6% (*p* = 0.044). Table 3 shows the description of healthcare pathway times. Medical coordination centre alert management time and times spent attending to patients remained unchanged, with a total median of the healthcare pathway of 81 min (IQR 31.2).

**Table 3:** Description of the healthcare pathway times

	Year 2019 Median (IQR)	Year 2020 Median (IQR)	*p*
Medical coordination centre times			
Alert-resource allocation time (min)	3.28 (2.33)	3.03 (2.51)	0.613
Resource allocation–activation time (min)	1.31 (1.22)	1.36 (1.09)	0.541
Total management response time (min)	5.16 (5.02)	5.04 (4.47)	0.737
Patient care time			
Time to reach the incident (min)	7.55 (6.41)	7.11 (5.30)	0.863
In situ healthcare time (min)	30.45 (15.16)	30.14 (16.24)	0.948
Time to reach the hospital (min)	14.49 (8.12)	13.20 (9.08)	0.672
Patient transfer time (min)	26.48 (12.04)	28.37 (13.38)	0.530
Total process time (min)	80.57 (31.32)	81.53 (29.40)	0.482

*IQR* interquartile range

### Trauma severity and clinical outcomes

More priority 0 and 1 patients were attended to during 2020, with a marked difference detected in priority 1 (*p* = 0.032). In these patients, a significant increase in mean age (46.6 years: SD 19.1; *p* = 0.002) was observed compared to the other groups.

**Fig. 2:**
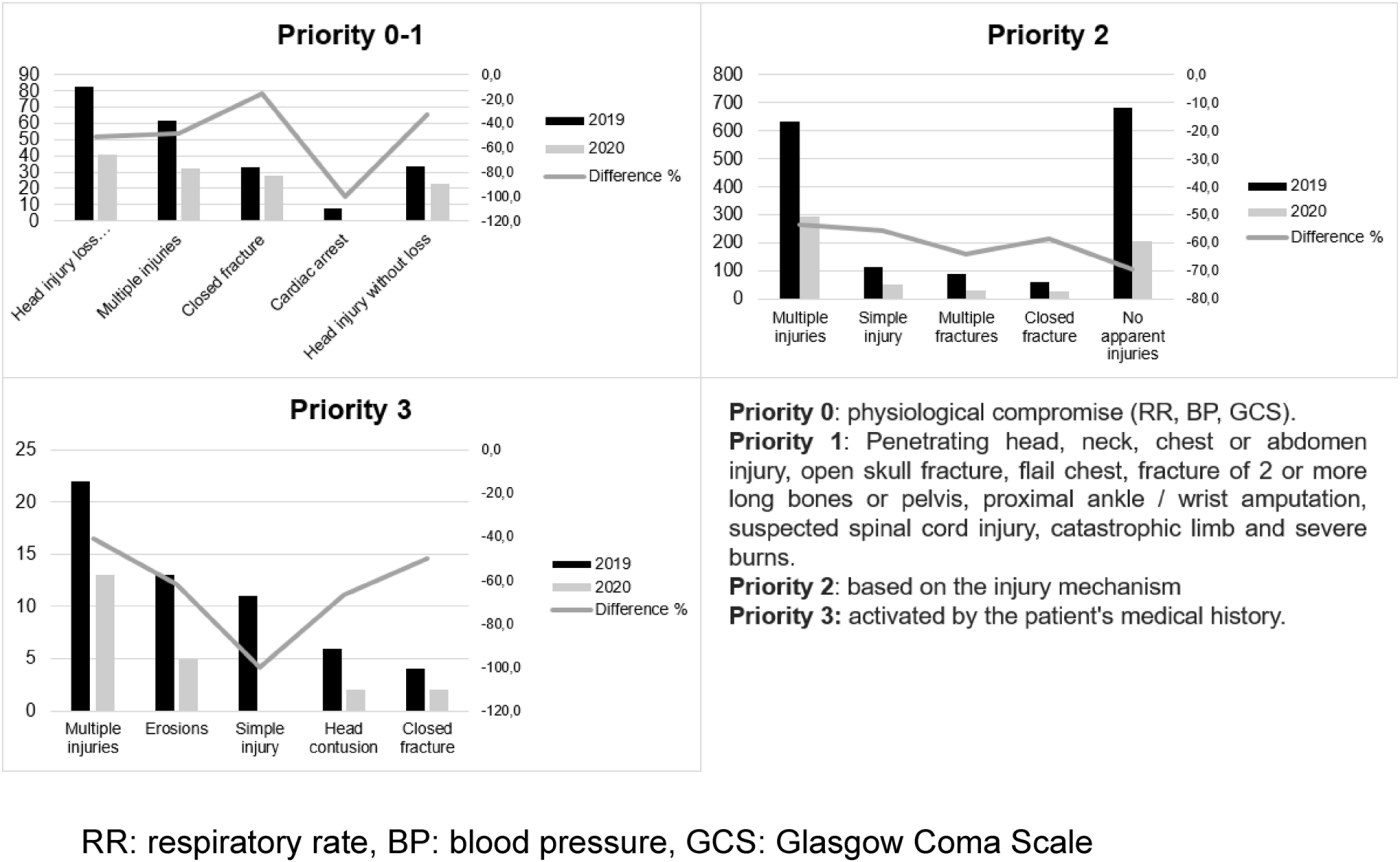
Description of diagnoses based on PTP code priority and difference between the two study periods. *RR* respiratory rate, *BP* blood pressure, *GCS* Glasgow Coma Scale

Limbs (53.1%), the head (37.6%) and thorax (22.5%) were the body areas most affected. Despite an increase in injuries to the head, spine and pelvis in 2020, no significant differences were detected. The most common primary diagnosis during the two periods was multiple injuries (34.4%) followed by head injury (22%), with no significant differences between years, as can be seen in Fig. 2.

Regarding respiratory status, 90.8% were not initially affected, and only 5.9% required invasive airway management. Haemodynamics behaved similarly, with no alterations in 88.7% of cases. In the period corresponding to 2020, no patients suffered trauma-based cardio-respiratory arrest, compared to eight in 2019 (0.4%). Concerning patients’ neurological status, measured by the Glasgow Coma Scale (GCS), 81.5% had a score of 15, although after lockdown lower GCS scores were recorded (84.4% with GCS < 15 prior to lockdown and 77.3% subsequently; *p* = 0.019).

For priority 0, the mean score on the Glasgow Coma Scale (GCS) was 8, whereas it remained at 15 for priorities 1–3. Comparative analysis does not reveal any differences, globally, for respiratory, haemodynamic or neurological status during the pandemic period (Table 2).

### Clinical management

There is decrease in the use of fluid therapy and peripheral intravenous access during the pandemic period (25.3% in 2020 vs 19.2% in 2021; *p* = 0.004). However, there were no significant changes in the number of cardiopulmonary resuscitation and oral intubation procedures in severe patients, who were all attended to by ALS units.

The most frequent prehospital treatment were splints (12.8%) and immobilizers (43.6%) with no statistical differences between the two periods. The application of tourniquets was extremely low, with a recorded use of 0.3% of the total (with no differences between the two periods).

### Hospital mortality

Finally, we analysed hospital mortality which, at 3.9% was unchanged over the two study periods (*p* = 0.328). The most common diagnosis associated with mortality was severe trauma brain injury (44.5%) and hypovolemic shock (21.6%), although there were no differences between periods.

## Discussion

The number of polytrauma patients treated in Catalonia decreased significantly during the COVID-19 pandemic. However, the severity of their injuries did not. Even during the post-lockdown period, from May 2020 onwards, the figures did not approach those of the previous year, in line with other studies that describe a fall of up to 44% [14]. Road traffic accidents, including vehicle–pedestrian collisions, decreased significantly due to the mobility restrictions in force during this period [15]. Such a decrease has also been described in the paediatric population in Canada, where injuries due to road traffic accidents virtually disappeared during the COVID period [16]. Despite the decrease in the number of injuries during the initial phase of the pandemic, patient severity increased. This has also been described in Germany [17]. The percentage of more severe physiological variables and critical anatomical areas, identified as priorities 0 and 1, increased.

Series published in the United Kingdom revealed a decline in general traumatology of up to 26%, but without undergoing an increase in the percentage of more severe patients [18]. The increase in the number of severe patients may be related to the greater occurrence of potentially severe injuries, such as injury to the head, pelvis and spine.

No differences are observed in patients’ epidemiological profile, the most frequent patient remaining a middle-aged male involved in a road traffic accident [19].

The first wave of the pandemic posed a challenge to the teams involved in prehospital care, as they had to adapt to the changing typology of the persons notifying and patients. An increase was observed in the allocation of BLS units to trauma patients. ALS units were increasingly occupied with providing home care to patients infected with SARS-CoV-2. This was also described in the research conducted by Laukkanen et al. [20].

There was a notable increase in patients treated for weapon wounds and burns, especially in the home, and an increasing incidence of traumatic falls. This increase in weapons wounds and burns in the home cannot be ruled out as being related to an increase in cases of gender-based violence [21]. Regarding the increase of fall patients during lockdown, its association with suicide attempts cannot be ruled out either [22, 23]. This may be associated with the increase in patients treated with mental health disorders during the study period, and hence further studies will be required. Further studies would require conducting and further evidence is needed.

Diagnoses have changed little for all priorities, with head injury involving loss of consciousness as the leading cause among the most severe patients (Fig. 2).

In respect of healthcare provision times, there were no differences between the two periods, which shows that, despite the complexity of providing healthcare, times attending to patients went unchanged. This key aspect was not observed in the provision of healthcare to time-dependent pathologies, such as stroke, which has undergone increased delays in first aid and increased complications [24]. Other studies on trauma patients focus on the impact of the pandemic on the reorganization undergone by hospitals, without addressing the prehospital phase of the healthcare pathway [25].

The main limitation of this study is that it only includes information supplied by prehospital care (EMS), apart from hospital mortality. We do not dispose of data on hospital length of stay, ICU admissions and hospital diagnoses upon discharge. Moreover, we cannot rule out selection bias, since patient inclusion depends on the implementation of the polytrauma code by the professional attending to the patient. However, its main strength is that we have collected data from all out-of-hospital polytrauma events occurring within Catalonia because the EMS covers the entire territory.

The study allows concluding that the pandemic caused a sharp decline in polytrauma events in our region, especially due to a decrease in the number of road traffic accidents as a result of mobility restrictions, and led to an increase in other types of accidents, mainly violent assaults, burns and falls. The profile of patients has changed as they present with increased severity and injuries to the head-spine and pelvis. Finally, despite the difficulties in attending to time-dependent pathologies, quality standards relating to the delay times throughout the prehospital healthcare pathway were maintained.
